# Calcium in Vascular Smooth Muscle Cell Elasticity and Adhesion: Novel Insights Into the Mechanism of Action

**DOI:** 10.3389/fphys.2019.00852

**Published:** 2019-08-07

**Authors:** Yi Zhu, Jing Qu, Li He, Feng Zhang, Zijing Zhou, Shanzhong Yang, Yong Zhou

**Affiliations:** ^1^Division of Pulmonary, Allergy and Critical Care Medicine, Department of Medicine, University of Alabama-Birmingham, Birmingham, AL, United States; ^2^Dalton Cardiovascular Research Center, University of Missouri-Columbia, Columbia, MO, United States; ^3^Department of Pathophysiology, School of Basic Medicine, Tongji Medical College, Huazhong University of Science and Technology, Wuhan, China; ^4^Department of Ophthalmology, The Second Xiangya Hospital, Central-South University, Changsha, China; ^5^Department of Respiratory Medicine, The Second Xiangya Hospital, Central-South University, Changsha, China

**Keywords:** vascular smooth muscle cell, calcium, elasticity, adhesion, extracellular matrix-integrin-cytoskeletal axis

## Abstract

Vascular smooth muscle cells (VSMCs) are the predominant cell type in the arterial wall. These cells play a critical role in maintaining vascular homeostasis including vasoconstriction and vasodilatation through active contraction and relaxation. Dysregulation of VSMC function alters the response of blood vessels to mechanical stress, contributing to the pathogenesis of vascular diseases, particularly atherosclerosis and hypertension. The stiffness of VSMCs is a major regulator of vascular function. Previous studies suggest that intracellular Ca^2+^ controls the stiffness of VSMCs by a mechanism involving myosin contractile apparatus. More recent studies highlight important functions of cytoskeletal α-smooth muscle actin (α-SMA), α5β1 integrin, and integrin-mediated cell-extracellular matrix (ECM) interactions in Ca^2+^-dependent regulation of VSMC stiffness and adhesion to the ECM, providing novel insights into the mechanism of calcium action.

## Introduction

Vascular smooth muscle cells (VSMCs) are mainly composed of the medial layer of the blood vessels, which are subjected to mechanical stress and pressure of blood flow, and maintain vascular tone and resistance. VSMCs not only have normal cellular functions, maintain their intrinsic properties, support vascular geometry, but also require some important actions to participate in the regulation of the biomechanical and biochemical properties of blood vessels (Li et al., 2018; Sanyour et al., 2018). In addition, VSMC alters vascular volume and local blood pressure and continuously regulates the physical and geometric shape of the blood chamber in response to blood pressure and mechanical stimulation (Hartman et al., 2016; Leloup et al., 2019).

VSMC carries out biomechanical and biochemical functions that are associated with the transformation of vascular configurations and are characterized by mechanical and physical effects such as blood vessel contraction and relaxation. Due to the various and diverse phenotypes of smooth muscle cells, these diversity effectively allow the blood vessels to gain flexibility and defend various physiological and pathological conditions (Yang et al., 2017; Hays et al., 2018).

VSMCs regulate the development and progression of vascular lesions, and the deep understanding of the relationship between vascular disease and mechanical stress provides some valuable data for the mechanical properties (e.g., elastic modulus, hardness) in those pathological processes. Currently, many publications contribute valuable data on the elastic changes of VSMC during physiology and disease processes and address some of the new concepts of cell elasticity in vascular disease (Sehgel et al., 2013; Sanyour et al., 2018, 2019). VSMCs are attached to the extracellular matrix (ECM) by interaction between some proteins (e.g., fibronectin, collagen) in the extracellular space and cell adhesion molecules (e.g., α5β1 integrin) that act as transmembrane proteins (Sehgel et al., 2015a,b; Zhang et al., 2016). The α5β1 integrin provides a mechanical link between the cytoskeleton (actin filament) component and the ECM, and actin responds to mechanical forces to the alternate cytoskeletal system of VSMC *via* integrin-mediated cell-ECM interaction (Zhou et al., 2017a,b). There is now ample evidence that the interactions between actin and integrin are responsible for reflecting and regulating the elasticity and adhesion characteristics of VSMC (Hong et al., 2015; Lacolley et al., 2017).

Calcium is a stimulator of many cellular functions, and under physiological conditions, intracellular calcium are present in VSMC to regulate many different biophysical and biochemical processes (Huang et al., 2018). The purpose of this review is to investigate the role of intracellular calcium in the regulation of VSMC elasticity and adhesion properties by influencing the ECM-integrin-cytoskeleton axis. This work summarizes a new concept that the increase in arterial stiffness is attributable to the dynamic changes of VSMC stiffness and adhesion process, opening up a new treatment for VSMC stiffness treatment of arteriosclerosis, and develops in-depth knowledge and insight into the dynamic behavior of calcium activity underlying cell hardness as well as the ability to correlate these mechanical events with ECM and cell adhesion interactions.

## Vascular Smooth Muscle Cell Stiffness and Adhesion

VSMC has a crucial function of maintaining vascular tone and resistance and involves all physiological and pathological changes that occur in the vessel wall. VSMCs are stromal cells of the blood vessel wall that are constantly exposed to mechanical signals and biochemical components produced in the blood compartment (Wang et al., 2015). The mechanical function of VSMCs relies on anchoring, requiring physical binding of cells to ECM and adjacent cells to perform force transfer functions associated with modulating vascular diameter (Zhu et al., 2018b). VSMC elasticity not only examines the biomechanical properties of whole cells, but also the biomechanical properties of subcellular structures such as microtubules, actin filaments, and intermediate filaments (Lacolley et al., 2018). The current study of VSMC elasticity supports a new concept that changes in VSMC stiffness, and oscillation are not only due to changes in ECM, but also due to changes in cytoskeletal actin filament content and arrangement, which are intrinsic conformations of VSMC (Hill and Meininger, 2016; Zhang et al., 2016). The cytoskeletal α-smooth muscle actin (α-SMA) is the actin isoform that predominates within VSMCs (Ye et al., 2014). Cytochalasin D (CD) is a drug that destroys the actin cytoskeleton network, and colchicine is another drug that depolymerizes microtubules, which are used to treat VSMCs. By CD evaluation, the α-SMA was shown to be the decisive cytoskeleton of the mechanical and elastic properties of VSMC (Zhu et al., 2012). Two forms of α-SMA appear within VSMCs. One form is a globular monomer molecule called G-actin, and the other form is a linear double-stranded filament polymer called F-actin, which is polymerized and organized by G-actin. Dynamical alternation between G-actin and F-actin constructs different high-level linkage structures in VSMC, affecting cell elasticity and cellular stress-relaxation behavior (Hong et al., 2012, 2014). Integrins are composed of two non-covalent associated α and β subunits, appear as heterodimeric transmembrane receptors for ECM proteins that mediate force transmission and signal transduction. Integrin-mediated adhesion of VSMCs to ECM proteins provides an important physical link between ECM and the cytoskeleton for bidirectional transmission of mechanical forces and production of biochemical cell signals through intracellular and extracellular mechanisms (Khalili and Ahmad, 2015; Tsai et al., 2018; Shen et al., 2019). The increasing arterial stiffness in arteriosclerosis, thrombosis, and age-related vascular disease was shown to enhance the expressions of α-actin and α5β1 integrin in VSMCs (Qiu et al., 2010; Laurent and Boutouyrie, 2015; DuPont et al., 2019). The α-SMA responds the bio-mechanical forces *via* integrin-mediated cell-ECM interactions to alternate cytoskeleton system of the cell. The α5β1 integrin works as the dominant fibronectin (FN) receptor to provide the bio-mechanical linkage between α-SMA and FN in extracellular space (Wu et al., 2010; Dhar et al., 2017). Inflexible VSMC stiffness and inefficient interaction with α5β1 integrin cause arterial stiffness to respond slowly and toughly to mechanical stresses from the blood flow. Conversely, flexible VSMC elasticity and active interaction with α5β1 integrin will efficiently respond to mechanical stress from the blood flow (Brozovich et al., 2016). The abnormal interaction between α5β1 integrin and α-SMA in VSMC is a main factor to cause the chaos of vascular tone and some vascular diseases such as hypertension, and VSMC is regulated by the cytosolic Ca^2+^ level and Ca^2+^ sensitivity of cytoskeletal filaments (Pierce, 2017).

## Calcium and ECM-Integrin-Cytoskeletal Axis

As one of the most popular second messengers, Ca^2+^ plays an important role in cell signaling and enters the cytoplasm through calcium channels on the membrane of VSMC (such as Ca^2+^ binding receptors and voltage-gated calcium channels) and some internal organelles (e.g., endoplasmic reticulum or mitochondria). In VSMCs, intracellular Ca^2+^ concentration ([Ca^2+^]_i_) is in a range of 100 to 10,000 nM, while Ca^2+^ concentration in extracellular space is 2 mM (Sun et al., 2017). Increasing [Ca^2+^]_i_ activates VSMC elastic and adhesive functions by way of two avenues: 1) entry of Ca^2+^ from extracellular space through specific ion channel, such as L-type Ca^2+^ channel (LTCC); 2) release of Ca^2+^ from intracellular organelles (sarco/endoplasmic reticulum) (Brozovich et al., 2016; Rodenbeck et al., 2017). [Ca^2+^]_i_ performs a major role in the regulation of VSMC elasticity and adhesion by influencing the ECM-integrin-cytoskeletal axis (Wu et al., 1998, 2001). The α-SMA of cytoskeleton importantly responds mechanical forces through integrin-mediated cell-ECM interactions to mediate VSMC stiffness and adhesive processes, and its activity and expression are modulated by [Ca^2+^]_i_. [Ca^2+^]_i_ regulates the α5 integrin subunit activity and expression, and the β1 integrin subunit is responsible to perform Ca^2+^ entry. [Ca^2+^]_i_ is a hub for the regulation of elasticity and adhesion of VSMC by influencing the ECM-integrin cytoskeletal axis to configure contractility and cell signaling (Huang et al., 2018). This information is important advancements in our understanding of VSMC stiffness and ECM adhesion coordination mechanisms.

### [Ca^2+^]_i_ Regulates Expression of α-SMA in VSMCs

**Figure 1:**
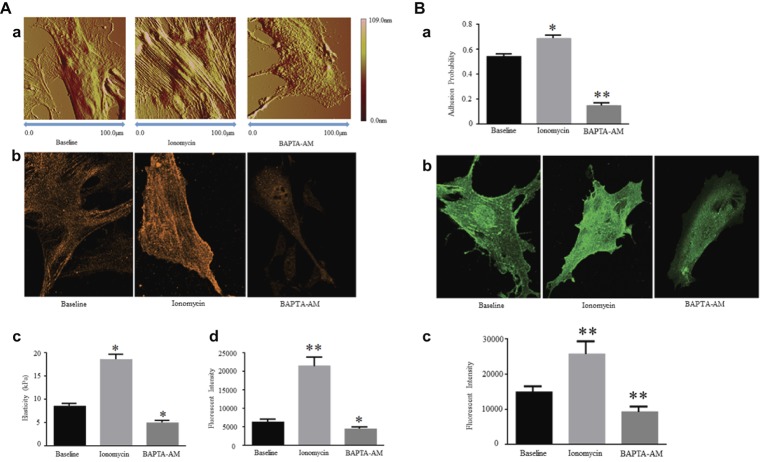
**(A)** [Ca^2+^]_i_ regulates Expression of α-SMA and cellular stiffness in VSMCs. Ionomycin increased [Ca^2+^]_i_, but BAPTA-AM decreased [Ca^2+^]_i_. **(a)** Atomic force microscopy topography analysis of VSMCs treated with or without drugs by increasing or decreasing [Ca^2+^]_i_. The VSMC area and height increase by a higher [Ca^2+^]_i_ level, and those decrease by a lower [Ca^2+^]_i_ level. **(b)** Atomic force microscopy stiffness analysis of VSMCs treated with or without drugs by increasing or decreasing [Ca^2+^]_i_. **(c)** Representative confocal immunofluorescent images showed α-SMA expression of VSMCs treated with or without drugs by increasing or decreasing [Ca^2+^]_i_. **(d)** α-SMA-positive signals among VSMCs treated with or without drugs by increasing or decreasing [Ca^2+^]_i_. Results are expressed as mean ± SEM, ^**^p < 0.01 and ^*^p < 0.05 vs. baseline. Reprinted from Zhu et al. (2018b) by published permission. **(B)** [Ca^2+^]_i_ regulates α5 integrin subunit expression and α5β1 integrin adhesion activities in VSMCs. Ionomycin increased [Ca^2+^]_i_, but BAPTA-AM decreased [Ca^2+^]_i_. **(a)** Atomic force microscopy measurement of probability of adhesion to FN matrix of VSMCs treated with or without drugs by increasing or decreasing [Ca^2+^]_i_. **(b)** Representative confocal immunofluorescent images showed α5 integrin expression of VSMCs treated with or without drugs by increasing or decreasing [Ca^2+^]_i_. **(c)** α5 integrin-positive signals among VSMCs treated with or without drugs by increasing or decreasing [Ca^2+^]_i_. Results are expressed as mean ± SEM, ^**^p < 0.01 and ^*^p < 0.05 vs. baseline. Reprinted from Zhu et al. (2018b) by published permission.

The current researches reported that the expression and production of α-SMA in VSMCs are regulated by [Ca^2+^]_i_ level. Ionomycin, a drug that increases [Ca^2+^]_i_ levels, was used to treat VSMCs and induce the increases in VSMC elasticity and adhesion activities, whereas they decreased after drug BAPTA-AM treatment to reduce [Ca^2+^]_i_. Ionomycin treatment was also observed the area and height of VSMCs to be expanded and increased due to developing cytoskeletal α-SMA production and promoting F-actin assembly. In contrast, BAPTA-AM decreased the area and height of VSMCs due to reducing cytoskeletal α-SMA production and preventing F-actin assembly (Zhu et al., 2018a) (Figure 1A, Reprinted from Zhu et al., 2018b by published permission).

Angiotensin II (ANG II) is a peptide chain and complicatedly converted by Angiotensin I, and Angiotensin II is found in VSMCs to stimulate the Gq protein and start a series of cascade reactions increasing [Ca^2+^]_i_ level (Meininger et al., 1991). ANG II generates to increase [Ca^2+^]_i_ by way of Gq protein pathway and induce a significant elevation in VSMC density and direction of actin stress fibers for promoting the VSMC stiffness (Hong et al., 2015).

If [Ca^2+^]_i_ exceeds the physiological requirement within VSMCs, it also will induce some vascular dysfunctions and diseases, such as hypertension. Hypertension is a common age-related vascular disease, and many factors can induce age-related vascular dysfunctions and diseases. However, a less efficiency of intracellular Ca^2+^ activity to attenuate the function of ECM-integrin-cytoskeletal axis regulating VSMC elasticity importantly contributes to induce hypertension (Touyz et al., 2018). With the age increasing, the regulative ability of cytosolic Ca^2+^ attenuates and makes VSMC to be stiffer and more adhesive to FN matrix, and the coordinate ability of integrin-actin reacts slowly due to a low intracellular Ca^2+^ signal transduction activity (Ghosh et al., 2017). VSMCs of old monkeys were shown a significantly increasing in cellular area and height in comparison to those of young counterparts. The VSMC area and height of spontaneously hypertensive rats (SHR) were also found a significant elevation in comparison to those of wild-type rats. The VSMC topography and shape were changed by α-SMA over production and F-actin over assembly due to a [Ca^2+^]_i_ signal transduction disorder by ECM-integrin-cytoskeletal axis in age-related vascular dysfunctions and diseases (Zhu et al., 2012; Sehgel et al., 2013).

An increase in aortic stiffness is often observed in people with hypertension, obesity and diabetes. In these vascular dysfunctions and diseases, the over production of α-SMA within VSMC is a decisive contributor to cause the increased aortic stiffness (Sehgel et al., 2013; Jia et al., 2015). Calcium channel blockers, such as benidipine and nifedipine, block Ca^2+^ influx flowing from the extracellular space through the specific ion channel to enter in the VSMC and reduce [Ca^2+^]_i_ for the treatment of hypertensive patients. [Ca^2+^]_i_ declination can promote the reduction of α-SMA expression and the breakdown of F-actin filaments in VSMCs, thereby reducing arterial stiffness and blood pressure (Miao et al., 2015; Yang et al., 2019). The drug thapsigargin inhibits the internal store (sarco/endoplasmic reticulum) Ca^2+^-ATPases to increase [Ca^2+^]_i_ and correspondingly enhances VSMC stiffness by developing the over expression of α-SMA (Cheng et al., 2019). These results indicated a high degree of correlation between the increase in [Ca^2+^]_i_ and the α-SMA expression and VSMC stiffness.

### [Ca^2+^]_i_ Regulates α5β1 Integrin Activity in VSMCs

Some observations supported a strong role for α5β1 integrin in increasing vascular tone and augmenting Ca^2+^ entry, whereas the role of αvβ3 integrin is present to reduce vascular tone and decrease Ca^2+^ entry. α5β1 integrin adheres to several major vascular ECM proteins, including fibronectin (FN), collagen I (COL-I), vitronectin (VN), and laminin I (LM), but the characteristics of adhesion to each protein are different with binding and signaling strongly associated with FN and COL-I (Sun et al., 2012; Hong et al., 2014, 2015). Previous application of blocking antibodies methods further identified that the interaction between FN and α5β1 integrin makes a specific and essential significance in the mechanical transduction of VSMC and confirmed α5β1 integrin to be the major receptor of FN (Sun et al., 2005, 2008; Wu et al., 2010). In integrin proteins, β1 and β3 subunits are responsible to perform a reverse function on Ca^2+^ entry. The mechanisms linking integrins to vascular control have not been entirely elucidated but some evidence showed that α5β1 and αvβ3 integrins link to the regulation of Ca^2+^ entry through the L-type Ca^2+^ channel (Meininger and Davis, 1992; Wu et al., 1998, 2001). Most importantly, inhibition of α5β1 integrin will decrease Ca^2+^ signaling in response vasoconstrictors but Ca^2+^ signaling will be unaffected or enhanced by αvβ3 inhibition (Janoštiak et al., 2014; Turner et al., 2015). The β1 subunit protein enhances Ca^2+^ signaling to reflect VSMC elastic characterization, and inhibition of β1 subunit will cause the declination of Ca^2+^ signaling for adhesion (Brozovich et al., 2016). Moreover, present researches verified that ionomycin increases [Ca^2+^]_i_ level to enhance the α5 integrin subunit expression in VSMCs and promote the adhesion activities to FN matrix. In verse, BAPTA-AM reduces the α5 integrin subunit expression and prevents the adhesion activities to FN matrix due to the lower [Ca^2+^]_i_ level (Zhu et al., 2018a) (Figure 1B, Reprinted from Zhu et al., 2018b by published permission). These data show that the α5β1 integrin is mainly responsible to act the VSMC adhesion to FN matrix *via* the regulation of [Ca^2+^]_i_ level. These results showed a high degree of correlation between the increase in [Ca^2+^]_i_ and the α5β1 integrin activity and adhesion to FN matrix.

**Figure 2:**
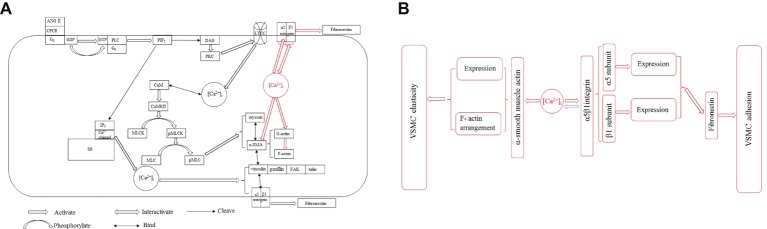
**(A)** Two pathways of [Ca^2+^]_i_ influencing VSMC stiffness and adhesion properties in VSMCs. [Ca^2+^]_i_-induced α-SMA and α5β1 integrin expressions for regulating VSMC stiffness and adhesion (red line) *via* ECM-integrin-cytoskeletal axis is parallel to the Ca^2+^-CaM-MLCK pathway in VSMCs (black line). All abbreviations in figure: [Ca^2+^]_i_, Intercellular Ca^2+^ concentration; ANG II, Angiotensin II; GPCR, G-protein-coupled receptor; G_q_, Guanine nucleotide-binding protein; GDP, Guanosine diphosphate; GTP, Guanosine triphosphate; G_α_, G protein α subunit; PLC, Phospholipase C; PIP_2_, Phosphatidylinositol 4,5-bisphosphate; IP_3_, Inositol trisphosphate; DAG, Diacylglycerol; SR, Sarcoplasmic reticulum; PKC, Protein kinase C; LTCC, L-type Ca^2+^ channel; CaM, Calmodulin; CaMKII, CaM-dependent protein kinase II; MLCK, Myosin light chain kinase; pMLCK, Phosphorylated myosin light chain kinase; MLC, Myosin light chain; pMLC, Myosin light chain phosphatase; α-SMA, α-smooth muscle actin; FAK, *Focal adhesion kinase*. **(B)** The new concept of [Ca^2+^]_i_ influencing VSMC stiffness and adhesion properties *via* ECM-integrin-cytoskeletal axis. [Ca^2+^]_i_ directly manipulates the expressions of α-SMA and α5β1 integrin, and the α5β1 integrin regulates [Ca^2+^]_i_ level in accordance with physiological requirement.

The intracellular Ca^2+^ is a central role to regulate the elasticity and adhesion of VSMC by configuring contractile and cellular signaling through the ECM-integrin cytoskeletal axis. The α-SMA of cytoskeleton is the major factor and responds mechanical forces through integrin-mediated cell-ECM interactions to mediate VSMC stiffness and adhesive processes, and its activity and expression are modulated by [Ca^2+^]_i_. [Ca^2+^]_i_ regulates α5β1 integrin activity and expression, and the β1 integrin subunit is responsible for Ca^2+^ entry. These information are important advances for us to understand the VSMC stiffness and ECM adhesion coordination mechanisms (Figure 2A, redline).

### [Ca^2+^]_i_ Influences VSMC Stiffness and Adhesion

Ca^2+^ influx flows from ion channel on the plasma membrane and the internal store (sarco/endoplasmic reticulum) to modulate a series of cellular events including VSMC elastic function *via* some enzymes’ phosphorylation. In VSMCs, G-protein-coupled receptor (GPCR) activates phospholipase C (PLC) and cleaves phosphatidylinositol 4,5-bisphosphate (PIP_2_) into diacylglycerol (DAG) and inositol trisphosphate (IP_3_) to form and participate Ca^2+^ activity. The IP3 diffuses to the sarcoplasmic reticulum (SR) and binds with a receptor on SR membrane to open a ligand-gated Ca^2+^ channel and increase [Ca^2+^]_i_. The DAG activates protein kinase C (PKC). The PKC often directly regulates and controls the opening and closing of L-type Ca^2+^ channel (LTCC) by phosphorylation and dephosphorylation, while indirectly manipulates VSMC elastic and adhesive functions by triggering [Ca^2+^]_i_ level (Okuyama et al., 2018; Zhang et al., 2018). Ca^2+^ is the loop in excitation-contraction coupling. The LTCC is a voltage-gated calcium channel and responsible for excitation-contraction coupling in VSMCs. Calmodulin (CaM) is a Ca^2+^-binding messenger protein to bind Ca^2+^ and transduce calcium signals performing its function. [Ca^2+^]_i_ binding with CaM activates CaM-dependent protein kinase II (CaMKII) to regulate the activation of myosin light chain kinase (MLCK) by phosphorylation and controls the interaction between α-SMA and myosin to affect the VSMC elasticity and adhesion process, while reducing the level of [Ca^2+^]_i_ by dephosphorylation inhibits the activity of MLCK to promote vascular smooth muscle relaxation (Snedden and Fromm, 2001; Martinsen et al., 2014). Ca^2+^ also retracts from cytoplasm to the internal store and extracellular space to decrease [Ca^2+^]_i_ level *via* the dephosphorylation of those enzymes, meanwhile inhibits myosin light chain phosphatase (pMLC) to hydrate the establishment with α-SMA (Kuo and Ehrlich, 2015; Saddouk et al., 2017). Many proteins involved in VSMC stiffness and adhesion including actin coordinating proteins are generated and driven by intracellular Ca^2+^, such as gelsolin, talin, vinculin, and cadherin (Martinez-Lemus et al., 2009; Hong et al., 2014). Gelsolin responds to the assembly and breakdown of actin filaments, and its activity is stimulated by [Ca^2+^]_i_ (Mikami et al., 2017). Cadherin responds VSMC adhesion and depends on [Ca^2+^]_i_ level (Desai et al., 2019). Vinculin is an actin binding protein and the bridge protein between talin and α-SMA, which is a target protein of PKC phosphorylation driven by [Ca^2+^]_i_ (Shen et al., 2019). Active intracellular Ca^2+^ induces to form the complex with FAK, talin, paxillin, and other components, and the complex enhances VSMC adhesion to FN matrix (Hong et al., 2015). The balance between phosphorylation and dephosphorylation for the activities of those enzymes is determined by the activity of intracellular Ca^2+^, and VSMCs are regulated by a complex network of phosphorylation and dephosphorylation kinase cascade from Ca^2+^ generation (Saddouk et al., 2017). Blebbistatin, which is a highly affinitive and selective inhibitor to myosin activity and phosphorylation, declines [Ca^2+^]_i_ activity for restraining vascular smooth muscle contraction to treat hypertension and some age-related vascular diseases (Zhang et al., 2017). The drug lysophosphatidic acid (LPA) enhances α5β1 integrin adhesion to FN matrix and activates Rho kinase to act as a signaling molecule and promote the level of [Ca^2+^]_i_ (Staiculescu et al., 2014), whereas the drug Y27632 inhibits the Rho-associated protein kinase (ROCK) to dephosphorylate and prevents α-SMA binding with myosin to decrease the need of [Ca^2+^]_i_ and attenuate the VSMC elasticity and adhesion (Zhou et al., 2017b). MLCK and pMLC were found to be over expressed in spontaneously hypertensive rats to stiffen the VSMC and attenuate the coordinate ability of ECM-integrin-actin owing to the low activity and oscillation behavior of [Ca^2+^]_i_ (Sehgel et al., 2013; Rodenbeck et al., 2017). ML7 is a MLCK inhibitor to treat the VSMCs exploring the role of a [Ca^2+^]_i_ dependent actin-myosin interaction and cause an reduction of VSMC stiffness and adhesion decreasing [Ca^2+^]_i_ level of VSMC (Zhu et al., 2012; Cheng et al., 2015; Huang et al., 2018). KCl was acted as a non-receptor agonist to elevate [Ca^2+^]_i_ and treated VSMCs to show a rapid transient increase in cell stiffness as well as cell adhesion to FN matrix due to transiently increasing in the level of [Ca^2+^]_i_, after KCl treatment the increased VSMC stiffness and adhesion to FN matrix were not abolished by MLCK inhibitor (Huang et al., 2018). These observations suggest that Ca^2+^-CaM-MLCK pathway in VSMCs is parallel to [Ca^2+^]_i_-induced α-SMA and α5β1 integrin expressions for regulating VSMC stiffness and adhesion (Figure 2A, blackline).

ATP-dependent Ca^2+^ pump and Na^+^/Ca^2+^ exchanger are two basic mechanisms for Ca^2+^ entry and exit from cells (Nance et al., 2015). These two mechanisms keep the balance of Ca^2+^ level between extracellular space and intracellular cytoplasm, and also keep the fluid and electrolyte balance of VSMCs to regulate cell normal physiological functions and elastic characters. The Na^+^/Ca^2+^ exchanger handles vascular tissues and blood pressure in human body through the regulation of VSMC elasticity (Ella et al., 2010). ATP-dependent Ca^2+^ pump and Na^+^/Ca^2+^ exchanger arise some damages of living organisms, and dehydration and overhydration will derive from the unbalances of electrolytes Na^+^, Ca^2+^, K^+^, Cl^−^, ${\mathrm{HPO}}_{3}^{2-}$, $H_{2}{\mathrm{PO}}_{3}^{-}$, ${\mathrm{PO}}_{4}^{3-}$, and Mg^2+^ (Cheng et al., 2019). If fluid and electrolyte unbalances happen, living organisms will be edema in vascular tissues. VSMCs lose the elastic characterization and adhesive interaction between ECM and cytoskeleton, and the diameters of blood vessels will be diminished and the flow blood meets a strong and vehement stress from a narrow blood lumen. The fluid and electrolyte unbalances also possibly cause the deposits of salt minerals, and many salt minerals are inside VSMC and disturb a lot of cellular functions including cell elasticity and adhesion (Ngai et al., 2018; Tangvoraphonkchai and Davenport, 2018). Additionally, ATP-dependent Ca^2+^ pump and Na^+^/Ca^2+^ exchanger occur some pathological damages to produce a lot of crisis and hardness affecting the entry and release of Ca^2+^ and making [Ca^2+^]_i_ declination. This declination will reduce Ca^2+^ signaling function for that phosphorylation and dephosphorylation activate or deactivate the corresponding enzymes to generate a cascade cycle for the modulation of VSMC elastic and adhesive characterizations. The abnormal Ca^2+^ release and entry promote pathological reactions to induce calcification, and the main form of calcification is calcium phosphate crystalline in VMSCs. Meanwhile, the produced calcium salts harden to lose the corresponding physiological mediation functions (Sun et al., 2017). Phosphorylation and dephosphorylation are the balance to activate some enzymes modulating a cascade cycle for Ca^2+^ regulation, but calcification cuts off all pathways and makes Ca^2+^ to fully lose its activity, and further induces VSMCs to thoroughly lose elastic and adhesive functions (Ngai et al., 2018). The calcification of VSMC is a very strongly risk factor to cause blood circulation morbidity and vascular mortality, and the detailed biochemical and pathological mechanisms are currently unknown (Leopold, 2015; McArthur et al., 2017).

## Conclusion

[Ca^2+^]_i_ directly manipulates the expressions of α-SMA and α5β1 integrin, and the α5β1 integrin regulates [Ca^2+^]_i_ level in accordance with physiological requirement (Figure 2B). This investigation is parallel to the Ca^2+^-CaM-MLCK pathway to influence VSMC stiffness and adhesion *via* ECM-integrin-cytoskeletal axis without any cascade cycles by phosphorylation and dephosphorylation. However, the physiological mechanism need to be further probed. This investigation factually plays an independent pathway to regulate VSMC stiffness and adhesion *via* ECM-integrin-cytoskeletal axis and provides a new therapy approach to target α-SMA or α5β1 integrin for influencing VSMC stiffness and adhesion and treat some vascular dysfunctions and diseases. Animal models provide important clues for exploring the pathogenesis of vascular dysfunctions and diseases to study how [Ca^2+^]_i_ to regulate and mediate VSMC elastic and adhesive functions in living organisms (Lacolley et al., 2014; Qiu et al., 2014). The performance at a single cell level is still a main research direction, and the observation will be focused on *in vivo* rather than *in vitro* because the normal physiological processes in living organisms provide some novel views to revolve [Ca^2+^]_i_ role in VSMC elastic and adhesive activities. In general, a more in-depth study of the [Ca^2+^]_i_ track and role in VSMC elastic and adhesive activities *in vivo* and *in vitro* for vascular physiological and pathological processes is the future extension and direction, and the significant advancements will contribute some novel clinic approaches to vascular disease.

## Author Contributions

YZhu drafted and designed manuscript. YZhu, JQ, LH, SY, and YZhou edited and revised manuscript. YZhu, FZ, and ZZ prepared figures. YZhu, JQ, LH, FZ, ZZ, SY, and YZhou approved final version of manuscript.

### Conflict of Interest Statement

The authors declare that the research was conducted in the absence of any commercial or financial relationships that could be construed as a potential conflict of interest.

## Abbreviations

ANG II: Angiotensin II
α-SMA: α-Smooth muscle actin
BAPTA-AM: 1,2-Bis(2-aminophenoxy)ethane-N,N,N′,N′-tetraacetic acid tetrakis (acetoxymethyl ester)
[Ca^2+^]_i_: Intercellular Ca^2+^ concentration
CaM: Calmodulin
CaMKII: CaM-dependent protein kinase II
CD: Cytochalasin D
COL-I: Collagen I
DAG: Diacylglycerol
ECM: Extracellular matrix
FAK: Focal adhesion kinase
FN: Fibronectin
GDP: Guanosine diphosphate
GTP: Guanosine triphosphate
GPCR: G-protein-coupled receptor
G_q_: Guanine nucleotide-binding protein
G_α_: G Protein α subunit
IP_3_: Inositol trisphosphate
LM: Laminin I
LPA: Lysophosphatidic acid
LTCC: L-type Ca^2+^ channel
MLC: Myosin light chain
MLCK: Myosin light chain kinase
ML7: Hexahydro-1-[(5-iodo-1-naphthalenyl)sulfonyl]-1*H*-1,4-diazepine
PIP_2_: Phosphatidylinositol 4,5-bisphosphate
PLC: Phospholipase C
PKC: Protein kinase C
pMLC: Myosin light chain phosphatase
pMLCK: Phosphorylated myosin light chain kinase
SHR: Spontaneously hypertensive rat
SR: Sarcoplasmic reticulum
VN: Vitronectin
VSMC: Vascular smooth muscle cell
Y27632: (1R,4r)-4-((R)-1-aminoethyl)-N-(pyridin-4-yl)cyclohexanecarboxamide.
